# Analysis of Repair Mechanisms following an Induced Double-Strand Break Uncovers Recessive Deleterious Alleles in the *Candida albicans* Diploid Genome

**DOI:** 10.1128/mBio.01109-16

**Published:** 2016-10-11

**Authors:** Adeline Feri, Raphaël Loll-Krippleber, Pierre-Henri Commere, Corinne Maufrais, Natacha Sertour, Katja Schwartz, Gavin Sherlock, Marie-Elisabeth Bougnoux, Christophe d’Enfert, Mélanie Legrand

**Affiliations:** a Institut Pasteur, INRA, Unité Biologie et Pathogénicité Fongiques, Paris, France; b Univ. Paris Diderot, Sorbonne Paris Cité, Cellule Pasteur, rue du Docteur Roux, Paris, France; c Institut Pasteur, Imagopole, Plate-Forme de Cytométrie, Paris, France; d Institut Pasteur, Centre d’Informatique pour la Biologie, Paris, France; e Department of Genetics, Stanford University, Stanford, California, USA; f Unité de Parasitologie-Mycologie, Service de Microbiologie clinique, Hôpital Necker-Enfants-Malades, Assistance Publique des Hôpitaux de Paris (APHP), Univ. Paris Descartes, Paris, France

## Abstract

The diploid genome of the yeast *Candida albicans* is highly plastic, exhibiting frequent loss-of-heterozygosity (LOH) events. To provide a deeper understanding of the mechanisms leading to LOH, we investigated the repair of a unique DNA double-strand break (DSB) in the laboratory *C. albicans* SC5314 strain using the I-SceI meganuclease. Upon I-SceI induction, we detected a strong increase in the frequency of LOH events at an I-SceI target locus positioned on chromosome 4 (Chr4), including events spreading from this locus to the proximal telomere. Characterization of the repair events by single nucleotide polymorphism (SNP) typing and whole-genome sequencing revealed a predominance of gene conversions, but we also observed mitotic crossover or break-induced replication events, as well as combinations of independent events. Importantly, progeny that had undergone homozygosis of part or all of Chr4 haplotype B (Chr4B) were inviable. Mining of genome sequencing data for 155 *C. albicans* isolates allowed the identification of a recessive lethal allele in the *GPI16* gene on Chr4B unique to *C. albicans* strain SC5314 which is responsible for this inviability. Additional recessive lethal or deleterious alleles were identified in the genomes of strain SC5314 and two clinical isolates. Our results demonstrate that recessive lethal alleles in the genomes of *C. albicans* isolates prevent the occurrence of specific extended LOH events. While these and other recessive lethal and deleterious alleles are likely to accumulate in *C. albicans* due to clonal reproduction, their occurrence may in turn promote the maintenance of corresponding nondeleterious alleles and, consequently, heterozygosity in the *C. albicans* species.

## IMPORTANCE

Recessive lethal alleles impose significant constraints on the biology of diploid organisms. Using a combination of an I-SceI meganuclease-mediated DNA DSB, a fluorescence-activated cell sorter (FACS)-optimized reporter of LOH, and a compendium of 155 genome sequences, we were able to unmask and identify recessive lethal and deleterious alleles in isolates of *Candida albicans*, a diploid yeast and the major fungal pathogen of humans. Accumulation of recessive deleterious mutations upon clonal reproduction of *C. albicans* could contribute to the maintenance of heterozygosity despite the high frequency of LOH events in this species.

## INTRODUCTION

*Candida albicans* is a quasi-obligate diploid yeast (1) whose 32 Mb genome is organized in eight pairs of chromosomes with, on average, one heterozygous position every ~250 bp (2–4). Genomic studies have shown that the *C. albicans* genome displays a high degree of plasticity. Indeed, aneuploidies, gross chromosomal rearrangements, and loss-of-heterozygosity (LOH) events of various lengths and locations were observed in both commensal and clinical isolates and upon commensalism or passage of a *C. albicans* laboratory strain in animal models (5, 6). Importantly, the ability of *C. albicans* to undergo genome rearrangements and its apparent tolerance of such changes can be critical for its survival upon exposure to changing conditions, such as antifungal treatments (2, 7–9). In this respect, LOH events contribute to the expansion of hyperactive mutations leading to antifungal resistance (10–14). More generally, allelic differences within a *C. albicans* strain can result in variations in gene expression or protein production or function (15, 16). Hence, LOH events in *C. albicans* have been associated with phenotypic variation, such as amino acid auxotrophy or drug sensitivity (17, 18), white-opaque switching upon mating-type-like locus homozygosis (19–21), and adaptation to growth on alternative carbon sources (22).

LOH events can arise from the mechanisms used by *C. albicans* in response to DNA double-strand breaks (DSBs) or can be the consequence of chromosome nondisjunction events during mitosis. Repair of DNA DSBs by gene conversion without crossover (GC) explains short-range LOH, while repair by either break-induced replication (BIR) or mitotic crossover (MCO) leads to LOH that extends from the DNA DSB site to the telomere. In the absence of DNA DSB repair or upon chromosome nondisjunction, segmental chromosome losses (SCL) or whole-chromosome losses (WCL) are observed and the loss of a chromosome is often followed by a reduplication event (23). Interestingly, LOH events in strains derived from the *C. albicans* SC5314 strain, from which the reference sequenced genome is derived, appear to be biased toward one of the two haplotypes for several chromosomes. For instance, Forche et al. (24) observed that homologous recombination-mediated LOH in progeny resulting from the *C. albicans* parasexual cycle (25) had a strong bias toward one of the two haplotypes for chromosome R (ChrR), Chr2, Chr4, Chr6, and Chr7. A similar bias was observed in a *C. albicans* *rad52*Δ/*rad52*Δ mutant that showed an increased frequency of spontaneous unidirectional LOH (26). The recent finding that *C. albicans* can exist in a haploid form also led to the observation that one of the two haplotypes for Chr3, 4, 6, and 7 and for most of Chr1 is never observed in the homozygous state under laboratory growth conditions (1). Finally, an investigation of the events associated with LOH at a specific locus on *C. albicans* Chr4 revealed that WCL events leaving haplotype B as the sole remaining haplotype were never observed (27). Taken together, these studies have led to the hypothesis that recessive lethal alleles are present on *C. albicans* chromosome homologs and prevent some LOH events from being detected.

DNA DSBs have been shown to be very potent initiators of recombination in yeast and other organisms and consequently of LOH (28, 29). The mechanisms by which DNA DSBs are repaired can greatly influence the nature of the LOH events that affect the *C. albicans* genome. In this respect, genotoxic agents have been used to trigger DNA breaks (30–32) and to study DNA repair mechanisms in *C. albicans* (33–37). However, the use of genotoxic agents (27, 35, 38, 39) or physical or chemical stresses known to induce LOH (27, 39, 40) does not allow precise control of the nature or the location of a DNA break. In order to circumvent this limitation, DNA DSB repair assays based on rare-cutting endonucleases such as I-SceI have been developed in many organisms (41–48).

Here, we show how the combination of I-SceI-induced DNA DSB and a recently developed LOH reporter system (27) allowed us to precisely study the mechanisms involved in DNA DSB repair at a specific genomic location in the *C. albicans* genome. Importantly, our detailed analysis of LOH events resulting from an induced DNA DSB in strain SC5314 allowed us to identify recessive deleterious alleles on *C. albicans* Chr4 haplotype B (Chr4B) that explain why haplotype A for this chromosome cannot be lost. Furthermore, we have expanded this work to clinical isolates of *C. albicans* by the identification of recessive lethal alleles on Chr5. Taken together, our results suggest that recessive deleterious alleles could play a role in the maintenance of heterozygosity in the *C. albicans* species.

## RESULTS

### Development and validation of an I-SceI-dependent DNA DSB-generating system in *C. albicans*.

To study the mechanisms involved in the repair of a single DNA DSB in *C. albicans*, we took advantage of the I-SceI meganuclease, an intron-encoded homing endonuclease isolated from the yeast *Saccharomyces cerevisiae*. I-SceI recognizes an 18 bp-long sequence (49, 50) absent from the *C. albicans* genome*.* We also used an LOH reporter system located at the *PGA59-PGA62* locus on Chr4 (27, 51) that consists of a combination of the use of flow cytometry and two fluorescent markers (see Fig. S1A in the supplemental material). Briefly, while one homolog of Chr4 carries the gene encoding the blue fluorescent protein (BFP), the other homolog harbors the gene encoding the green fluorescent protein (GFP) (see Fig. S1A). Hence, LOH events can be detected by flow cytometry, as cells that have undergone an LOH at this locus express either the BFP or the GFP (see Fig. S1B). Further characterization of the LOH can be achieved either by single nucleotide polymorphism (SNP) typing or by whole-genome sequencing (WGS) after cell sorting (27, 52).

**FIG 1:**
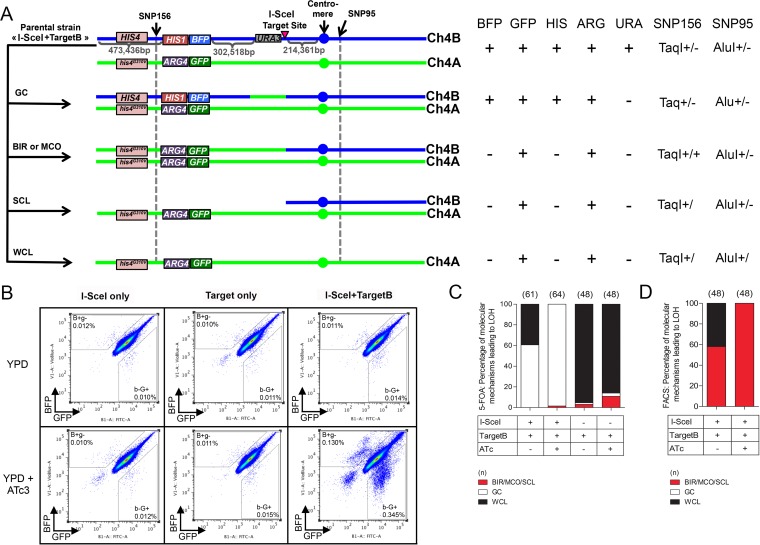
Effect of I-SceI induction in the I-SceI+TargetB and control strains. (A) Different LOH events on Chr4B can arise upon I-SceI induction. The heterozygous SNPs used for RFLP characterization are indicated with black arrows. SNP156, close to the left-arm telomere (at position 367295 on Chr4A and position 367352 on Chr4B), is part of a TaqI restriction site, while SNP95, on the right arm of Chr4 and close to the centromere (at position 1310251 on Chr4A and position 1310274 on Chr4B), is located in an AluI restriction site. One haplotype carries the restriction site, while the other does not. The combined heterozygosity or homozygosity of these SNPs gives insights about the molecular mechanisms leading to LOH events. The *HIS4* gene presenting a nonfunctional allele on haplotype A as shown by Gomez-Raja et al. (17) is also represented here. GC, gene conversion; BIR, break-induced replication; MCO, mitotic crossover; SCL, segmental chromosome loss; WCL, whole-chromosome loss. BIR/MCO and SCL events are indistinguishable. (B) Co-occurrence of I-SceI and its target sequence triggers a predominant increase in mono-GFP cells. The cultures were analyzed on a MACSQuant cytometer. Data represent 10^6^ events. The B^+^g^−^ and b^−^G^+^ gates were defined arbitrarily. (C and D) SNP-RFLP analysis shows that I-SceI-dependent DNA DSBs on Chr4B are mainly repaired by GC. Histograms present the proportion of BIR/MCO or SCL, GC, and WCL events in the population having undergone a LOH and recovered either from 5-FOA counterselection (C) or from FACS analysis (D). BIR/MCO or SCL events correspond to mono-GFP cells that displayed a homozygous SNP156 but have maintained a heterozygous SNP95. WCL events correspond to mono-GFP cells in which both SNP95 and SNP156 became homozygous. GC events correspond to doubly fluorescent cells in which both SN156 and SNP95 remained heterozygous.

We thus generated a *C. albicans* strain that carries (i) a tetracycline-inducible, codon-optimized gene encoding the rare-cutting endonuclease I-SceI modified to harbor the simian virus 40 (SV40) nuclear localization signal (NLS) (42, 53–55), (ii) a gene encoding a tetracycline-dependent transactivator (56), (iii) the I-SceI target sequence along with the *URA3* marker on the left arm of Chr4, and (iv) the fluorescence-activated cell sorter (FACS)-optimized LOH reporter system with the *BFP* gene linked to the *HIS1* gene on the left arm of Chr4B which also bears a functional allele of the *HIS4* gene closer to the telomere (17) and the *GFP* gene linked to the *ARG4* gene on the left arm of Chr4A which bears the nonfunctional *his4^G310V^* allele closer to the telomere (see Fig. S1C in the supplemental material) (17, 27, 51). In this setting, the I-SceI target sequence is ~215 kb distant from the Chr4 centromere, while the LOH reporter system is ~300 kb further toward the telomere (Fig. 1A). The resulting strain is referred to as “I-SceI+TargetB,” as WGS showed that the I-SceI target site and the *URA3* gene were inserted on Chr4B. WGS also showed that the I-SceI+TargetB strain had not experienced gross chromosomal rearrangements (aneuploidies, LOH) upon the occurrence of the successive transformation events needed for its construction (data not shown). Control strains lacking the I-SceI gene or the I-SceI target sequence were designated “Target only” and “I-SceI only,” respectively, and were used to assess the occurrence of I-SceI-independent DNA DSBs at the I-SceI target site or I-SceI-induced Chr4 off-target DNA DSBs.

In the I-SceI+TargetB strain, induction of the I-SceI gene through addition of a tetracycline derivative should result in I-SceI endonuclease production and targeting to the nucleus, followed by the generation of a DNA DSB at the I-SceI target sequence (see Fig. S1 in the supplemental material). DNA DSBs can be repaired either by GC, thus leading to doubly fluorescent cells that have lost the *URA3* gene (Ura^−^) and are 5-fluoroorotic acid (5-FOA) resistant (57), or by BIR/MCO leading to the loss of the *URA3* gene and BFP reporter and, thus, to the appearance of 5-FOA-resistant (5-FOA^r^), arginine prototroph (Arg^+^), histidine auxotroph (His^−^), mono-GFP cells (27, 51) (Fig. 1A). WCL/SCL should also lead to progeny with uridine and histidine auxotrophies and GFP fluorescence (Fig. 1A). Importantly, since the *HIS1* gene is linked to the *BFP* gene and all mono-GFP cells should be histidine auxotrophs, an unexpected crossover between the BFP/GFP locus and the heterozygous *HIS4*/*his4^G310V^* locus should not impact the phenotypes of cells that have undergone BIR/MCO, SCL, or WCL (Fig. 1A).

To validate the functionality of the I-SceI system, the I-SceI+TargetB and control strains were grown in the presence or absence of anhydrotetracycline (3 µg/ml; ATc) and plated on YPD (1% yeast extract, 2% peptone, 2% dextrose) and 5-FOA agar plates (27). For both control strains, no increase in the number of 5-FOA^r^ colonies was observable under the noninduced and induced conditions, with a rate below 1.0 × 10^−7^ events/cell/generation under both conditions (Table 1). In contrast, I-SceI expression yielded a 372-fold increase in the rate of appearance of 5-FOA^r^ colonies compared to the noninduced condition using the I-SceI+TargetB strain (Table 1).

**TABLE 1:** 5-FOA resistance quantification on Chr4

Strain	Growthcondition	5-FOA^r^ acquisition rate (×10^−8^)^a^(no. of events/cell/generation)	Foldchange^a^
I-SceI+TargetB	YPD	11	372
	YPD + ATc	4,100	
I-SceI+TargetA	YPD	0.4	2,450
	YPD + ATc	980	
I-SceI+TargetA+*GPI16*	YPD	2.44	574
	YPD + ATc	1,400	
I-SceI only	YPD	6.1	1.2
	YPD + ATc	7.4	
Target only	YPD	6.3	0.5
	YPD + ATc	3.4	

a Values are representative of results of 2 independent experiments.

The I-SceI+TargetB and control strains were also grown for 8 h in the presence or absence of ATc and analyzed by flow cytometry. This allows detecting long-range LOH events only, i.e., BIR, MCO, SCL, or WCL. As expected, a 30-fold ATc-dependent increase in the mono-GFP frequency was observed for the I-SceI+TargetB strain and no change was detected in the controls (Fig. 1B and Table 2), consistent with the I-SceI recognition sequence being located on the BFP-bearing chromosome, Chr4B. Additionally, an increase was noticeable in the number of nonfluorescent cells, likely to be dead cells, as previously shown by Loll-Krippleber et al. (27). Strikingly, we also observed a 17-fold increase in the frequency of appearance of mono-BFP cells in the ATc-treated I-SceI+TargetB cells only (Fig. 1B and Table 2). The basis for this unexpected population of monofluorescent cells is revisited below.

**TABLE 2:** LOH quantification on Chr4 by flow cytometry

Strain	Cellpopulation	Growthcondition	*n^a^*	LOH frequency± SEM (×10^−4^)	Foldchange	Mann-Whitneytest *P* value
I-SceI+TargetB	Mono-GFP	YPD	37	1.35 ± 0.1	30	≤0.0001
		YPD + ATc	40	40.0 ± 0.9		
	Mono-BFP	YPD	37	1.0 ± 0.1	17	≤0.0001
		YPD + ATc	40	16.8 ± 0.6		
I-SceI+TargetA	Mono-GFP	YPD	36	2.0 ± 0.1	9	≤0.0001
		YPD + ATc	36	18.7 ± 0.3		
	Mono-BFP	YPD	36	1.8 ± 0.1	48	≤0.0001
		YPD + ATc	36	85.5 ± 1.6		
I-SceI+TargetB+*GPI16*	Mono-GFP	YPD	36	0.3 ± 0.02	105	≤0.0001
		YPD + ATc	36	31.6 ± 2.2		
	Mono-BFP	YPD	36	0.5 ± 0.04	58	≤0.0001
		YPD + ATc	36	29.0 ± 1.3		
I-SceI+TargetA+*GPI16*	Mono-GFP	YPD	36	0.3 ± 0.02	43	≤0.0001
		YPD + ATc	35	14.8 ± 0.9		
	Mono-BFP	YPD	36	1.0 ± 0.1	100	≤0.0001
		YPD + ATc	35	101.8 ± 1.6		
I-SceI only	Mono-GFP	YPD	21	1.1 ± 0.1	1	0.9424
		YPD + ATc	21	1.1 ± 0.1		
	Mono-BFP	YPD	21	1.0 ± 0.0	1	0.4605
		YPD + ATc	21	0.9 ± 0.0		
Target only	Mono-GFP	YPD	21	1.0 ± 0.1	1	0.0514
		YPD + ATc	21	0.9 ± 0.1		
	Mono-BFP	YPD	21	1.1 ± 0.1	1	0.2251
		YPD + ATc	21	1.0 ± 0.1		

a Each value represents the number of biological replicates analyzed.

Taken together, these results indicated that I-SceI is functional and induces a target-specific DNA DSB in *C. albicans*. In addition, the different increases in the frequencies of 5-FOA^r^ (372-fold, including both long- and short-range LOH events) and mono-GFP (30-fold, including long-range LOH events only) cells upon induction of the I-SceI gene suggested that long- and short-range LOH events occur at different frequencies.

### I-SceI-induced DNA DSBs are predominantly repaired by GC.

5-FOA^r^ colonies obtained following I-SceI induction can arise from point mutation in the *URA3* gene or as a consequence of DNA DSB-triggered GC, BIR, MCO, or WCL/SCL events (Fig. 1A) (23). We used PCR to assess whether 5-FOA resistance was a consequence of *URA3* loss or point mutation and SNP typing to assess the heterozygous or homozygous state of SNPs of interest, allowing to deduce the length of the LOH and to distinguish between GC, BIR, MCO, and WCL/SCL events (52) as illustrated in Fig. 1A. We used SNP156 (TaqI restriction site on the left arm of Chr4A), located between the telomere and the BFP/GFP locus, and SNP95 (AluI restriction site on the right arm of Chr4A), located close to the centromere (52). PCR of 64 5-FOA^r^ clones derived from strain I-SceI+TargetB revealed that none of these clones had acquired 5-FOA resistance by point mutation in the *URA3* gene, as *URA3* itself could no longer be detected. Furthermore, SNP156 and SNP95 remained heterozygous in 98.5% (63/64) of the tested clones, consistent with a GC event. The remaining clone was homozygous for SNP156 and heterozygous for SNP95, suggesting a BIR/MCO/SCL event. Consistently, all 5-FOA^r^ clones with GC-mediated LOH events were still expressing both BFP and GFP and were His^+^ Arg^+^ whereas the unique 5-FOA^r^ clone with a BIR/MCO/SCL-mediated LOH event was expressing GFP only and was found to be His^−^ Arg^+^.

To assess whether the high number of GC-mediated LOH events was specifically linked to I-SceI expression, we tested 61 5-FOA^r^ colonies arising from the I-SceI+TargetB strain grown in the absence of ATc and from the Target only strain that lacks the I-SceI gene. We found that in the I-SceI+TargetB strain, 60.7% of the 5-FOA^r^ clones (37/61) had undergone LOH through GC and 39.3% (24/61) through WCL (Fig. 1C) in the absence of ATc. When testing Target only 5-FOA^r^ clones, WCL appeared as the main mechanism leading to LOH events (95.4%) on Chr4 in both the absence and the presence of ATc. Differences observed between the results obtained for the I-SceI+TargetB strain grown in the absence of ATc and the Target only strain might reflect leakage of the P_*TET*_ promoter in the absence of inducer. Thus, taken together, our results indicated that GC is the predominant mechanism for the repair of an I-SceI-induced DNA DSB on Chr4B in *C. albicans.*

In order to determine the frequency with which BIR/MCO, SCL, and WCL might occur when GC was not the mechanism of repair, we used FACS analysis to isolate the mono-GFP cells observed by flow cytometry that were likely to have undergone long-range LOH events. A total of 48 confirmed mono-GFP clones were analyzed for the loss of auxotrophic markers, SNP156 homozygosity, and SNP95 heterozygosity. All tested clones were Ura^−^ His^−^ Arg^+^. As expected, SNP typing revealed that all clones obtained from the induced culture had repaired the I-SceI-induced DNA DSB by BIR, MCO, or SCL (Fig. 1D). Furthermore, genome sequencing of a subset of these clones identified no cases of SCL (data not shown). In contrast, under the noninduced conditions, LOH events were the result of BIR, MCO, or SCL (58%) but also WCL (42%) (Fig. 1D).

Taken together, our results revealed that a majority of *C. albicans* cells repaired an I-SceI-induced DNA DSB on Chr4B by GC but that BIR or MCO could also be used, although at lower frequency.

### GC-independent repair of an I-SceI-induced DNA DSB on Chr4A leads to inviable progeny.

**FIG 2:**
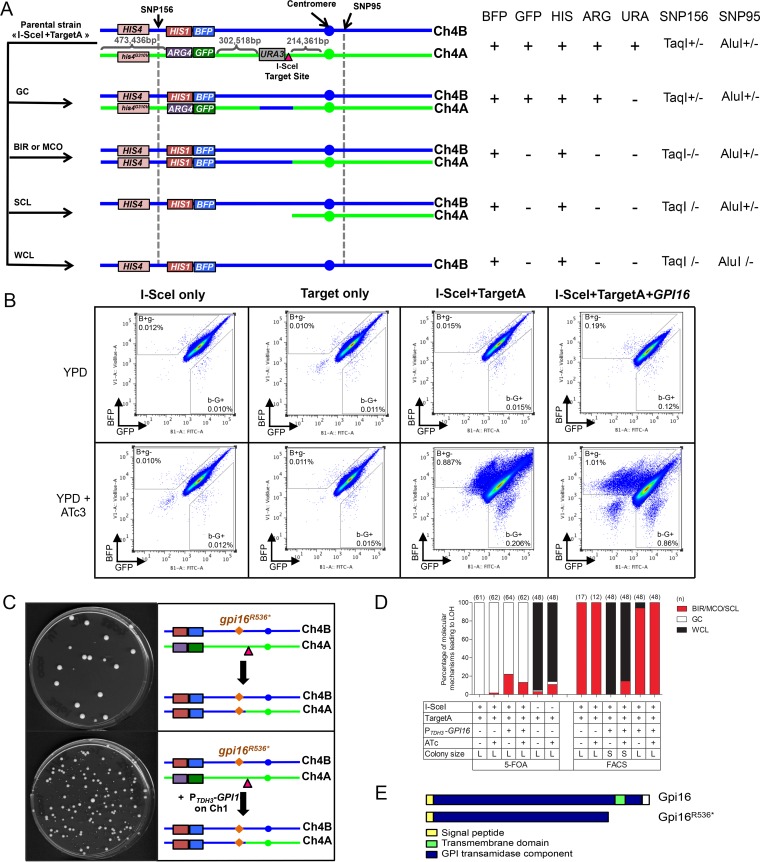
Effect of I-SceI induction in the I-SceI+TargetA and control strains. (A) Different LOH events on Chr4A can arise from I-SceI. As seen in Fig. 1A, the combined heterozygosity or homozygosity of SNP156 and SNP95 gives insights about the molecular mechanisms leading to LOH events. GC, gene conversion; BIR, break-induced replication; MCO, mitotic crossover; SCL, segmental chromosome loss; WCL, whole-chromosome loss. (B) Co-occurrence of I-SceI and its target sequence triggers a predominant increase in levels of mono-BFP cells. Data represent 10^6^ events. The B^+^g^−^ and b^−^G^+^ gates were defined arbitrarily. (C) Integration of the full-length allele of *GPI16* on Chr1 allows recovery of viable mono-BFP cells after cell sorting. While cells obtained from strain I-SceI+TargetA showed poor viability due to homozygosis of the gpi^R536*^ allele, complementation with a wild-type *GPI16* allele in strain I-SceI+TargetA+*GPI16* restored viability. The largest colonies observed in both cases were doubly fluorescent, having not undergone I-SceI cleavage on Chr4A. (D and E) SNP-RFLP analysis showed that I-SceI-dependent DNA DSBs on Chr4A are mainly repaired by GC. Histograms present the proportion of BIR/MCO or SCL, GC, and WCL events in the population having undergone a LOH recovered either from 5-FOA counterselection (D) or from FACS analysis (E). BIR/MCO or SCL events correspond to mono-BFP cells that displayed a homozygous SNP156 but have maintained a heterozygous SNP95. WCL events correspond to mono-BFP cells in which both SNP156 and SNP95 became homozygous. GC events correspond to doubly fluorescent cells in which both SN156 and SNP95 remained heterozygous. L, large-sized colonies; S, small-sized colonies. (F) The gpi16^R536*^ allele might result in the truncation of the Gpi16 protein carboxy-terminal transmembrane domain, part of the conserved GPI transamidase domain.

The results presented above were obtained with a strain that harbored the I-SceI target site on Chr4B. We and others have shown that LOH events on Chr4 are biased toward haplotype A, suggesting that Chr4B may bear one or more recessive lethal alleles that could influence the frequency with which DNA DSB repair mechanisms are detected in our assay (1, 24, 26, 27). Therefore, we constructed the “I-SceI+TargetA” strain, which carries the I-SceI target site on the GFP-bearing Chr4A homolog (Fig. 2A). WGS of I-SceI+TargetA confirmed the location of the I-SceI site on Chr4A, and gross chromosomal rearrangements were not observed (data not shown). We observed that growth of strain I-SceI+TargetA in the presence of ATc resulted in a large increase (2,450-fold) in the number of 5-FOA^r^ clones compared to the noninduced condition (Table 1). In addition, we observed a 48-fold increase in the number of mono-BFP cells upon induction. Again, we also observed an unexpected 8-fold increase in the number of cells expressing only the other fluorescent protein (mono-GFP) in the induced cultures of the I-SceI+TargetA strain (Fig. 2B and Table 2) (see below for further investigation of this observation).

SNP typing of 62 5-FOA^r^ clones revealed that 98.4% (61/62) arose from a GC event, in agreement with the cells being doubly fluorescent and His^+^ Arg^+^ (Fig. 2A and D). Hence, GC also appears to be the predominant mechanism for the repair of an I-SceI-induced DNA DSB on Chr4A in *C. albicans*. Unexpectedly, the remaining 5-FOA^r^ clone appeared to be mono-GFP by flow cytometry. This clone was homozygous for SNP156 but heterozygous for SNP95 and His^+^ Arg^−^. This suggested that this 5-FOA^r^ clone belonged to the population of rare mono-GFP cells observed by flow cytometry as described above and which are likely to have arisen by other recombination events (see below).

In order to determine the frequency of the molecular mechanisms giving rise to mono-BFP cells, we enriched them by FACS analysis and plated them onto YPD medium. Strikingly, only a subset of the plated cells was able to form colonies (~4%; Fig. 2C); characterization of the colonies highlighted two populations: (i) doubly fluorescent and His^+^ Arg^+^ Ura^+^ colonies—suggesting that they were wild-type cells, illegitimately recovered in our sorting procedure—and (ii) mono-BFP and His^+^ Arg^−^ Ura^+^ colonies—which likely resulted from an I-SceI-independent LOH (Fig. 2E). This result suggested that all mono-BFP cells that had arisen by repair of the I-SceI target site on Chr4A were inviable, possibly due to homozygosis of one or more recessive lethal alleles on Chr4B.

### A heterozygous null mutation in the *GPI16* gene is responsible for the inviability of *C. albicans* cells that are homozygous on the left arm of Chr4B.

Results presented above implied the presence of at least one recessive lethal allele on Chr4B between the left-arm telomere (position 1) and the I-SceI target site (position 778082). We reasoned that (i) the genotype for this allele should be heterozygous in *C. albicans* strain SC5314, as homozygosis of the Chr4A allele is viable; (ii) the recessive lethal allele should never be found in the homozygous state in the *C. albicans* population; and (iii) the allele should not affect a gene previously shown to be dispensable in *C. albicans*. In order to identify such an allele, we took advantage of sequencing data obtained from 155 *C. albicans* isolates, including the SC5314 reference strain (M.-E. Bougnoux, G. Sherlock, N. Sertour, K. Schwartz, C. Maufrais, and C. d’Enfert, unpublished data) and searched for SNPs generating a stop codon in open reading frames (ORFs) on Chr4B and meeting the criteria given above. Strikingly, only one such SNP was identified in SC5314, at position 659191 on Chr4B (equivalent to position 659155 on Chr4A), which resulted in a change from CGA (arginine) on Chr4A to TGA (stop codon) on Chr4B in the C4_03130W gene. The premature stop codon resulted in a protein that was shorter by 87 amino acids, deleting a C-terminal membrane-spanning domain (Fig. 2F). C4_03130W is the ortholog of the essential *S. cerevisiae GPI16* gene encoding a membrane-bound component of a glycosylphosphatidylinositol (GPI) transamidase complex necessary for GPI anchor biosynthesis. Notably, no disruptant could be obtained for the C4_03130W gene (now referred to as *GPI16*), suggesting that this gene is essential in *C. albicans* (58). Thus, our results suggested that the truncated *GPI16* allele (referred to as gpi16^R536*^) might be responsible for the inviability of *C. albicans* cells that experienced a long-range LOH on Chr4B.

**FIG 3:**
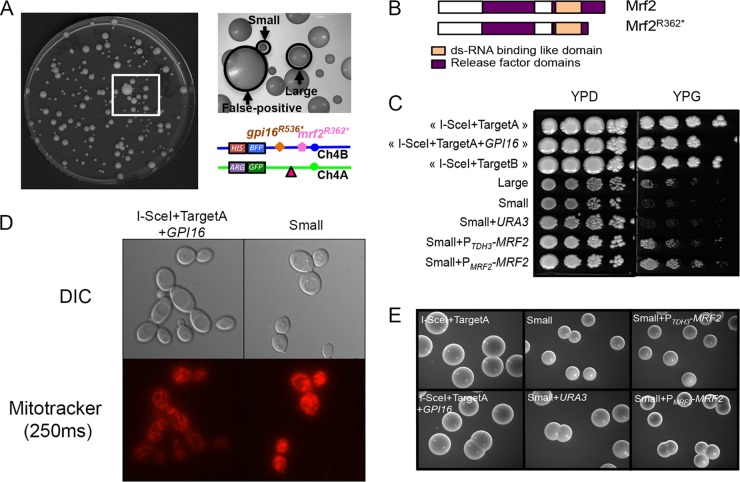
Homozygosis of Chr4B is associated with phenotypic heterogeneity due to an additional recessive deleterious allele. (A) Heterogeneous colony sizes of mono-BFP cells derived from the I-SceI+TargetA+*GPI16* strain. Small and large colonies are indicated by black arrows. Small-colony variants mainly resulted from WCL, while large-colony variants resulted from BIR/MCO. Very large colonies (false positive) are doubly fluorescent, having not undergone I-SceI cleavage on Chr4A. The locations of the mrf2^R362*^ and gpi16^R536*^ mutated alleles on Chr4B on both sides of the I-SceI target site are shown. (B) Homozygosis of mrf^R362*^ allele gives rise to a truncated nonfunctional protein. The Mrf2 protein encoded by the *MRF2* functional allele is 396 amino acids long, but when encoded by the mrf^R362*^ allele, if translated, the protein would be shorter by 34 amino acids, removing the C-terminal part of the release factor domain. (C) Small colonies have a respiratory defect that is restored upon complementation with *MRF2*. Cells were spotted on rich medium containing glucose or glycerol as the carbon source. No growth was observed on YPG for small colonies. Complementation with P*_TDH3_*-*MRF2* or P*_MRF2_*-*MRF2* restored growth on YPG, while complementation with *URA3* only did not. (D) Cells from the small colonies show a defect in the mitochondrial network. Cells were stained with MitoTracker for mitochondria. The panels display microscopy pictures of the control strain (I-SceI+TargetA+*GPI16*) and the small-colony-variant-derived cells. The cells were observed at ×100 magnification in differential inference contrast (DIC) and Cy3 for Mitotracker staining (250 ms—red). The cells were examined under a Leica DMRXA microscope. (E) Complementation with an *MRF2* functional allele does not restore wild-type colony size in small-colony variants on YPD agar medium. Pictures were taken with a Leica M80 stereomicroscope at a zoom of ×7.5.

To test this hypothesis, the *GPI16* wild-type ORF available in the *C. albicans* ORFeome (56) was placed under the control of the constitutive P_*TDH3*_ promoter and integrated at the *RPS1* locus on Chr1 in the I-SceI+TargetA strain, yielding strain I-SceI+TargetA+*GPI16*. As observed for the I-SceI+TargetA strain, growth of the I-SceI+TargetA+*GPI16* strain resulted in a 100-fold increase in the number of mono-BFP cells in the presence of ATc (Fig. 2B and Table 2). Interestingly, FACS-treated mono-BFP cells derived from strain I-SceI+TargetA+*GPI16* grown in the presence or absence of ATc showed 100% viability on YPD agar plates, although variability in colony size was observed with 54% of small and 46% of large colonies (Fig. 2C and 3A). These results contrasted with those obtained for strain I-SceI+TargetA and indicated that overexpression of *GPI16* complemented the inviability of mono-BFP cells. This confirmed that the gpi16^R536*^ allele was the recessive lethal allele responsible for this inviability.

As done previously, we evaluated the nature and frequency of the molecular mechanisms at the origin of mono-BFP cells derived from the I-SceI+TargetA+*GPI16* strain. As we observed variability in colony size, we independently analyzed 48 large and 48 small colonies. On the basis of auxotrophy, SNP typing, and WGS, we could conclude that the large colonies had all arisen by BIR/MCO while the small colonies had arisen predominantly by WCL and reduplication (85.4%). The remaining 14.6% small-colony variants had arisen by BIR/MCO. Notably, even though a crossover between the BFP/GFP locus and the heterozygous *HIS4*/*his4^G310V^* could have arisen and could have allowed the occurrence of mono-BFP cells with histidine auxotrophy, these were never observed.

The results presented above suggested that mono-BFP cells arose from an I-SceI-dependent DNA DSB that either was repaired by BIR/MCO or resulted in a WCL. As we observed that 46% of colonies were large and 54% were small among 1,054 FACS-sorted mono-BFP cells from strain I-SceI+TargetA+*GPI16* upon I-SceI induction, BIR/MCO and WCL seemed to have occurred at similar frequencies. However, homozygosis of a second recessive allele located between the I-SceI target site and the right telomere of Chr4B could explain the small-colony phenotype associated with Chr4B WCL (Fig. 3A).

### A heterozygous null mutation in the *MRF2* gene is partially responsible for the small-colony variants arising upon Chr4A loss.

Small colonies were predominantly observed upon Chr4A WCL under both noninduced and induced conditions (89/96 when pooled). We thus used the approach presented above to identify a recessive allele of a nonessential gene in the region of Chr4 extending from the I-SceI target site location to the right telomere responsible for the observed phenotype. Only one SNP was identified in SC5314, located at position 796698 on Chr4B (796679 on Chr4A), and it resulted in a change from CGA (arginine) on Chr4A to TGA (stop codon) on Chr4B in the C4_03750C gene (mrf2^R362*^) (Fig. 3A). The premature stop codon resulted in a protein that was shorter by 34 amino acids (Fig. 3B). This gene is the ortholog of *S. cerevisiae MRF1*, which encodes a putative mitochondrial translational release factor. Deletion of *MRF1* in *S. cerevisiae* and in other organisms results in acute respiratory defects (59–61), but the consequence of inactivating the C4_03750C gene in *C. albicans* has not yet been investigated. As the name *MRF1* has been assigned to the C1_11700C gene in *C. albicans* (62), an ortholog of *S. cerevisiae ETR1*, we instead refer to C4_03750C as *MRF2*.

In order to test whether mrf2^R362*^ was associated with respiratory defects in *C. albicans*, we first grew small-colony variants obtained from strain I-SceI+TargetA+*GPI16* on YPD and on YPG (1% yeast extract, 2% peptone, 2% glycerol, 2% agar) agar plates. Because glycerol is a nonfermentable carbon source, functional mitochondria are required for its assimilation via respiration. We observed that small-colony variants could not grow on YPG (Fig. 3C), in contrast to the parental strain and the large-colony variants derived from this strain, thus suggesting that the mrf2^R362^* allele is associated with mitochondrial dysfunction. Mitochondrion staining of cells derived from the small-colony variants and the I-SceI+TargetA+*GPI16* parental strain reinforced our hypothesis. Indeed, while the parental strain’s mitochondria appeared as an interconnected filamentous network, a characteristic of healthy cells, those of the small-colony variants appeared patchier (Fig. 3D). We further confirmed that the mrf2^R362^* allele was responsible for the mitochondrial defect, as complementation with the wild-type *MRF2* allele restored growth of the small-colony variants on YPG medium (Fig. 3C). Yet the colonies remained small on YPD medium (Fig. 3E), suggesting the occurrence of a third recessive deleterious allele on Chr4B, though we were unable to identify it.

### GC and CO are also involved in the repair of I-SceI-induced DNA DSBs.

**FIG 4:**
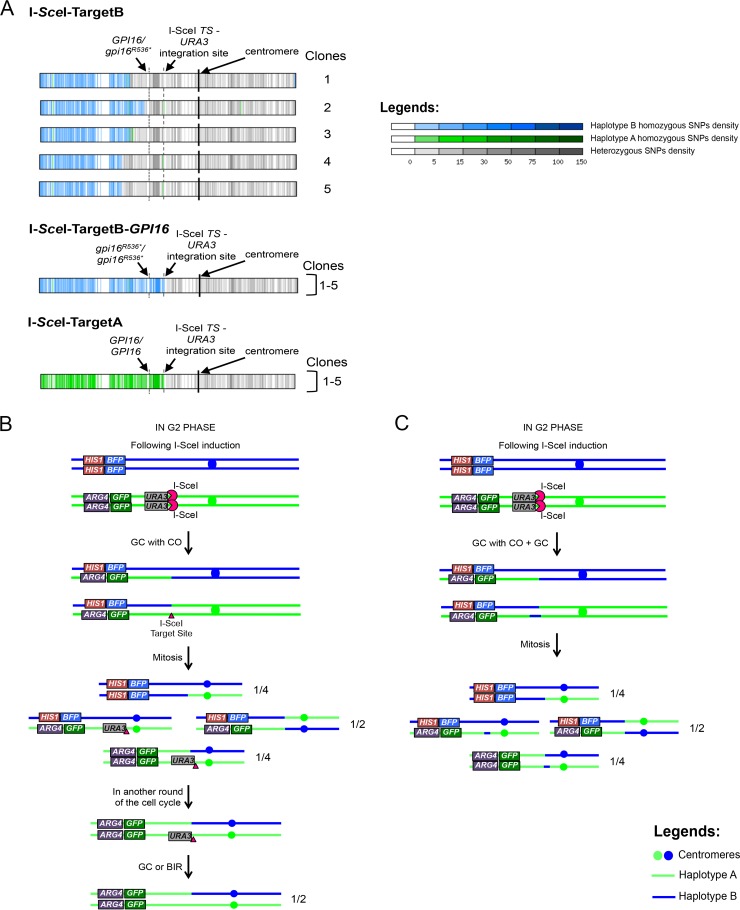
Unexpected LOH events result from independent BIR/MCO and GC events or GC with CO events. (A) Representation of LOH events that occurred in sequenced unexpected monofluorescent cells. WGS allowed the identification of LOH events occurring in the unexpected (i) mono-BFP cells from the I-SceI+TargetB strain; (ii) mono-BFP cells from the I-SceI+TargetB+*GPI16* strain (clone 5 displays an aneuploidy on Chr5; data not shown); (iii) mono-GFP from the I-SceI+TargetA strain (clone 4 displays a truncation of Chr3; data not shown). Chr4 for each strain (or group of strains) is represented as a horizontal box with vertical bars corresponding to 1-kb regions. Vertical bars are colored gray if heterozygous and green if haplotype A homozygous and blue if haplotype B homozygous (haplotypes A and B harbor the GFP and BFP genes, respectively). Different levels of gray, green, or blue intensity indicate local changes in SNP density. White regions are homozygous in both the sequenced strain and strain SC5314 (used to define haplotypes A and B). Centromeres are shown as black vertical bars. The locations of the *GPI16* and I-SceI target site-*URA3* loci are indicated. (B) Multiple but not simultaneous repair events could be responsible for the unexpected mono-GFP cells upon repair of an I-SceI-induced DSB in the I-SceI+TargetA strain. Upon I-SceI induction, one of the two chromatids is cut and repaired using the homologous chromosome as a template by GC with CO. After a mitotic event, one-quarter of the population became mono-GFP, with one homolog still carrying the *URA3* marker and I-SceI target sequence. Because both the *URA3* marker and I-SceI target sequence were found to be absent, we propose that, during the 8-h induction, cells having inherited the GFP-bearing chromosomes with one copy of *URA3* and the target site can go through another loop of DNA DSB repair in G_2_ phase, yielding 50% of the newly generated mono-GFP lacking the *URA3* marker or the target sequence. (C) Multiple and simultaneous repair events could alternatively be responsible for the unexpected mono-GFP cells upon repair of an I-SceI-induced DNA DSB in the I-SceI+TargetA strain. Upon I-SceI induction, both sister chromatids are cut: while one chromatid is repaired using the homologous chromosome as a template by GC with CO, the second sister chromatid is repaired by GC using the homologous chromosome as a template. After mitosis, one-quarter of the population has become mono-GFP. Models shown in panels B and C are also valid for mono-BFP cells from I-SceI+TargetB+*GPI16*.

As mentioned previously, induction of I-SceI expression yielded monofluorescent cells with unexpected fluorescence. Mono-BFP cells were observed upon the expression of I-SceI in the I-SceI+TargetB strain, while only mono-GFP cells were expected upon repair of the I-SceI-induced DNA DSB (Fig. 1B). Similarly, mono-GFP cells were observed upon I-SceI induction in the I-SceI+TargetA strain (Fig. 2B). To understand the basis for these rare cell populations, we first collected mono-BFP cells obtained from the I-SceI+TargetB strain after I-SceI induction. Almost all of the mono-BFP cells were inviable on YPD upon cell sorting (~96%), leading us to hypothesize that they had undergone a LOH event that had rendered Chr4B homozygous. Among the few viable cells, 12 mono-BFP cells had likely repaired the I-SceI-induced DNA DSB, as deduced from the loss of both the *URA3* gene and the I-SceI target sequence. These cells were analyzed by SNP typing and appeared to have undergone a BIR/MCO or SCL event, rendering SNP156 homozygous for haplotype B despite the presence of the I-SceI target sequence on the Chr4B homolog. We further investigated the nature of these events by WGS of 5 mono-BFP clones. As diagrammed in Fig. 4A, all 5 clones had undergone homozygosis, which had rendered Chr4 homozygous for haplotype B from the left telomere to a position to the left of position 659191 (<659191), i.e., upstream of the *GPI16* locus, instead of position 778082, where the I-SceI target site had been inserted. Hence, in these clones, heterozygosity was maintained in the region that encompasses the *GPI16* gene. We conclude that the viable mono-BFP cells arising from the I-SceI+TargetB strain were the result of two independent recombination events: an I-SceI-dependent GC event on Chr4B at the I-SceI-*URA3* locus and an I-SceI-independent BIR/MCO event on Chr4A that had led to the homozygosity of the left arm of Chr4B while preserving a functional *GPI16* allele.

On the basis of the gpi16^R536^* findings determined in the I-SceI+TargetA strain, we hypothesized that the inviability of most mono-BFP cells obtained from the I-SceI+TargetB strain was the result of homozygosis of the gpi16^R536^* allele. Thus, we generated strain I-SceI+TargetB+*GPI16* by integrating the *GPI16* wild-type allele placed under the control of the P_*TDH3*_ promoter at the *RPS1* locus on Chr1 in the I-SceI+TargetB strain. When I-SceI expression was induced in this strain, we observed a 58-fold increase in the appearance of mono-BFP cells (Table 2). This increase is 3 times higher than the frequency of unexpected mono-BFP cells in strain I-SceI+TargetB but can be explained by an increased viability of the cells during the time of the experiment. As we predicted, all mono-BFP cells recovered by FACS analysis were now viable. WGS of 5 mono-BFP clones revealed that they had undergone a LOH rendering Chr4 homozygous for haplotype B from the I-SceI-*URA3* locus to the left telomere (Fig. 4A). As illustrated in Fig. 4B and C, we hypothesize that these mono-BFP cells arose through successive or simultaneous repair events involving GC with CO followed by GC/BIR or MCO at the I-SceI sites.

Similarly, we collected unexpected mono-GFP cells obtained from the I-SceI+TargetA strain. These cells showed 100% viability, and those that had repaired the I-SceI-induced DNA DSB, as deduced from the loss of the *URA3* gene, appeared to have experienced BIR/MCO or SCL events. WGS of 5 mono-GFP cells confirmed that they had undergone homozygosis, rendering Chr4A homozygous from the I-SceI-*URA3* locus to the left telomere (Fig. 4A). Here again, we hypothesize that these mono-GFP cells arose through successive or simultaneous repair events involving GC with CO followed by GC/BIR or MCO at the I-SceI sites (Fig. 4B and C).

Taken together, these results suggested that, in addition to GC and BIR events, the repair of an I-SceI-induced DNA DSB on Chr4 could involve GC with CO events.

### Heterozygous mutations are also responsible for haplotype-specific LOH in clinical strains.

**FIG 5:**
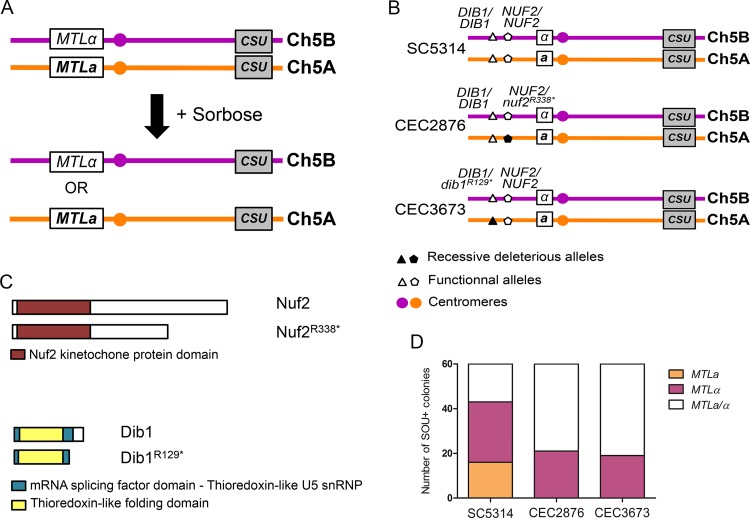
Heterozygous deleterious recessive alleles are also present in clinical isolates. (A) Principle of sorbose-induced WCL impacting Chr5 in *C. albicans*. *C. albicans* cells that have undergone Chr5 loss can grow on sorbose. Conveniently, Chr5 is heterozygous at the mating-type locus (*MTL****a***/α) and the heterozygous or homozygous state of this locus can be screened by PCR. (B) Map of the Chr5 showing the localization of the mutated alleles in clinical isolates. Applying the same method as for strains I-SceI+TargetA and I-SceI+TargetB, we identified a mutation in *NUF2* and *DIB1*, respectively, found in CEC2876 and CEC3673 clinical strains. These mutations are both localized on Chr5A. (C) Homozygosis of nuf2^R338*^ or dib1^R129*^ allele gives rise to truncated nonfunctional proteins. The *NUF2* and *DIB1* functional alleles encode proteins that are 470 and 149 amino acids in length, respectively, but when encoded by the nuf2^R338*^ and dib1^R129*^ alleles, the proteins are shorter by 142 and 29 amino acids, respectively. (D) Growth on sorbose gave rise to both *MTL**a*** and *MTL*α homozygous clones for SC5314 but only *MTLα* homozygous clones for the clinical isolate. The Chr5 loss was assessed through PCR at the *MTL* locus. While both homozygous *MTL* loci can be found for SC5314 reference strain, the presence of the mutated alleles at the heterozygous state on Chr5A prevents the homozygosis of this haplotype in both clinical isolates.

The identification of the recessive deleterious mutations gpi16^R536^* and mrf2^R362*^ was made in *C. albicans* strain SC5314. Notably, both mutations appeared unique to this strain. Therefore, we asked whether different recessive lethal alleles might occur in other *C. albicans* isolates. To this end, we focused on Chr5, as selection for utilization of l-sorbose as the sole carbon source by *C. albicans* has been reported to trigger the loss of one Chr5 homolog and, thus, whole Chr5 homozygosis (22, 63, 64). Chr5 also carries on its left arm the mating type-like locus (MTL), often found to be heterozygous (*MTL****a***/α) in *C. albicans* strains, which can be used as a marker of homozygosity upon genomic rearrangement on Chr5 (Fig. 5A). We hypothesized that if a *C. albicans* isolate were harboring a recessive lethal allele on Chr5, l-sorbose-utilizing (SOU^+^) progeny would undergo LOH events, maintaining only one of the two Chr5 haplotypes and thus a unique mating type. To test this hypothesis, we scanned the genomes of *C. albicans* isolates and identified strains CEC2876 and CEC3673 as harboring a heterozygous SNP on Chr5 that might generate a potentially nonfunctional allele of a presumably essential gene. In strain CEC2876, the identified SNP was located at position 289097 on Chr5A (289095 on Chr5B) and resulted in a change from CGA (arginine) on Chr5B to TGA (stop) on Chr5A in the C5_01280C gene (nuf2^R338*^). The premature stop codon resulted in a protein shorter by 132 amino acids. This gene is the ortholog of *S. cerevisiae NUF2*, which encodes a kinetochore component (Fig. 5B and C). In strain CEC3673, the identified SNP was located at position 212951 on Chr5A (212941 on Chr5B) and resulted in a change from AGA (arginine) on Chr5B to TGA (stop) on Chr5A in the C5_00920W gene (dib1^R129*^). The premature stop codon resulted in a protein that was shorter by 20 amino acids. This gene is the ortholog of *S. cerevisiae DIB1*, which plays a role in mRNA splicing and DNA methylation regulation (Fig. 5B and C). Deletion of either *NUF2* or *DIB1* in *S. cerevisiae* results in lethality, but the consequence of inactivating these ORFs in *C. albicans* has not yet been investigated.

After selection on sorbose-containing media, the heterozygous status of the *MTL* locus of 60 single SOU^+^ colonies was analyzed by PCR and SC5314 was used as a control. While isolates homozygous for both mating types that do not carry the *nuf2^R338^* and dib1^R129*^ alleles (16 *MTL**a*** and 27 *MTL*α) were recovered for SC5314 (Fig. 5D), only *MTL*α SOU*^+^* derivatives were recovered for CEC2876 and CEC3673 (21 *MTL*α and 19 *MTL*α, respectively) (Fig. 5D). In all three cases, the remaining progeny were found to maintain heterozygosity at the mating type-like locus, suggesting that they had acquired sorbose resistance independently of an LOH event encompassing the *MTL*, *DIB1*, and *NUF2* loci (Fig. 5D).

Taken together, these results indicated the occurrence of recessive lethal alleles in the heterozygous state in *C. albicans* isolates that were responsible for the haplotype bias observed when these isolates undergo LOH.

## DISCUSSION

Having a dynamic genome is now recognized as one of *C. albicans*’ characteristics that has led to its success as both a commensal and a pathogen. Yet little is known about the molecular events that lead to the genome rearrangements that are observed in *C. albicans* isolates. Our work aimed at studying the repair of DNA DSBs and facilitating the study of genome dynamics in *C. albicans*. Here, we have (i) developed a DNA DSB-inducing system that generates a DNA DSB at a defined site in the *C. albicans* genome; (ii) demonstrated that this induced DNA DSB is mainly repaired by gene conversion; (iii) explained the previously observed bias in Chr4 haplotype homozygosis by the presence of recessive lethal and deleterious alleles at the *GPI16* and *MRF2* loci in *C. albicans* strain SC5314; (iv) shown that similar haplotype biases occur in other *C. albicans* strains, due to different recessive lethal alleles; and (v) observed rare and complex molecular mechanisms involved in DNA DSB repair in *C. albicans*. This report presents for the first time a precise study of haplotype-dependent repair mechanisms in a natural heterozygous diploid organism.

### Mechanisms of double-strand-break repair in *C. albicans.*

In this study, we estimated the LOH frequency associated with I-SceI-induced DNA DSB by making use of two assays: 5-FOA counterselection, observed upon short- and long-range LOH events, and flow cytometry, whereby loss of either the BFP or GFP reveals long-range LOH only. Taken together, our results show that LOH events associated with the repair of a site-specific DNA DSB in *C. albicans* are due mainly to GC but also, at a low rate, to BIR/MCO. Notably, the locations of the I-SceI target site on Chr4A and on Chr4B had different outcomes with respect to the frequency with which BIR/MCO and WCL events were observed, with BIR/MCO and WCL occurring at similar frequencies when the target site was located on Chr4A whereas only BIR/MCO events were observed when the target site was located on Chr4B. We propose that the presence of different DNA conformations at the time of repair (65, 66) or of heterozygous alleles on one of the homologs could explain the observed homolog specificity of the molecular mechanisms resulting in LOH (27). Noticeably, our study did not assess nonhomologous end joining (NHEJ) repair events; however, NHEJ is thought to be inefficient for DNA DSB repair in *C. albicans* (33, 67).

Our work also highlighted a substantial fold increase of an unanticipated population of monofluorescent cells following an I-SceI-induced DNA DSB. Two hypotheses may explain this cell population. First, the cell population could result from the DNA DSB being repaired by GC with CO in the G_2_ phase (Fig. 4B). As a consequence, because I-SceI expression is induced for 8 h and the monofluorescent cells resulting from GC with CO would still carry the I-SceI target site, it is conceivable that cells could undergo a second I-SceI-mediated DNA DSB repaired by GC, BIR, MCO, or SCL that would be associated with the loss of the *URA3* marker (Fig. 4B). Alternatively, as suggested by others (68–70), I-SceI cleavage might occur in early S phase, when DNA is the most accessible (71), and therefore might happen concomitantly on the two sister chromatids. One sister chromatid could be repaired by a classical GC, while the other sister chromatid would undergo a GC with CO (Fig. 4C). Interestingly, Esposito and colleagues (72) reported that spontaneous GC events occur at a rate of 10^−7^ to 10^−6^ in diploid *S. cerevisiae*, while Haber and Hearn (73) quantified the occurrence of GC with CO events as 12% to 25% of the overall GC events in the presence of large homologous regions. More recently, researchers have found that the frequencies of GC events occurring with CO increased upon generation of a DNA DSB (74), supporting the results found in *C. albicans*.

Finally, our results also suggested the occurrence of an additional rare cell population arising from multiple events on Chr4: the repair of the I-SceI-induced break by GC followed by a spontaneous LOH event on Chr4A. This scenario would result in two homozygous regions: (i) one short tract of homozygosity surrounding the I-SceI target site and the *URA3* marker and (ii) one larger homozygous region starting upstream of the I-SceI target site and extending toward the left telomere (Fig. 4A). We are aware that our analyses using SNP-restriction fragment length polymorphism (SNP-RFLP) and WGS remain descriptive and are not a proof of the molecular mechanisms at the origin of the observed LOH events.

Taken together, our results have shown that DNA DSB repair in *C. albicans* most often involves GC. GC also appears to be the main repair mechanism of DNA DSBs generated by homing endonucleases (29, 75), more specifically, I-SceI (49), in *S. cerevisiae* and in other organisms (70, 76). Interestingly, Forche et al. (39) showed that stresses (H_2_O_2_, fluconazole, 39°C) affected the nature and/or frequency of LOH in *C. albicans*. In the absence of stress, or in the presence of H_2_O_2_, GC and BIR were observed as the main mechanisms leading to LOH events at the *GAL1* locus on Chr1 (39). However, under conditions of oxidative stress, an increase in the frequency of GC events and a decrease in the frequency of BIR events were observed. Yet these experiments could not provide information about haplotype specificity. Our approach of generating a targeted DNA DSB allows discrimination of the impacts of all of the haplotypes on LOH and the identification of both frequent and rare repair events associated with DNA DSBs. Nonetheless, our experiments have been carried out using a single locus for DNA DSB induction on Chr4 and our results could be locus specific; additional experiments should be conducted to extend our study to other chromosomes and loci. Additionally, although we show that I-SceI can be used in *C. albicans* to generate targeted DNA DSBs, the *C. albicans*-optimized clustered regularly interspaced short palindromic repeat (CRISPR)-Cas9 system (77) could help extend this study, as it allows targeting haplotype-specific targets without the sophisticated genome engineering that we had to implement.

### Recessive deleterious alleles in the *C. albicans* genome and their impact on LOH.

In this study, we have identified two recessive alleles present in the heterozygous state on Chr4B. The gpi16^R536*^ truncated allele was associated with lethality when found in the homozygous state and was therefore identified as responsible for unidirectional LOH on Chr4. We also identified a truncated allele of the nonessential *MRF2* gene. Complementation of the mrf2^R362*^/mrf2^R362*^ strains with a functional allele of *MRF2* restored mitochondrial function but did not impact colony size. This reflects (i) the presence of a third recessive allele on haplotype B that we were not able to identify or (ii) the presence of a heterozygous or homozygous recessive allele in the *C. albicans* genome that, upon homozygosis of mrf2^R362*^ mutated allele, leads to the small-colony phenotype or (iii) the possibility that, if translated, the nonfunctional Mrf2^R362*^ protein would have a dominant-negative effect. Nonetheless, the latter hypothesis does not explain why such a dominant-negative effect is not naturally observed in the heterozygous SC5314 strain, unless we take into consideration the difference in genome locations which could impact the level of expression of the functional copy of *MRF2*, inserted at the *RPS1* locus, compared to that of the endogenous *MRF2* locus.

Although our DNA DSB-inducing system, combined with the availability of a large panel of genome sequences for *C. albicans* isolates, allowed us to identify the mutation underlying the haplotype bias observed upon Chr4 homozygosis (1, 24, 26, 27) and a deleterious allele responsible for respiratory defects, our study was performed *in vitro* and we cannot rule out the existence of additional recessive alleles that would have deleterious effects *in vivo* when present in the homozygous state. In addition, our identification of the gpi16^R536*^ and mrf2^R362*^ alleles was unique to the SC5314 reference strain. Nevertheless, we also demonstrated that haplotype bias upon LOH of Chr5 can be found in two clinical strains, suggesting that our findings may extend to the entire *C. albicans* population.

In this study, only deleterious recessive alleles located on Chr4 of strain SC5314 could be revealed. However, haplotype bias has been observed for other chromosomes in this strain (part of Chr1, Chr3, Chr6, and Chr7) (1). Similarly to our observation with Chr4, it is likely that deleterious and possibly lethal recessive alleles are located on these chromosomes and responsible for these haplotype biases.

*C. albicans* reproduction has been shown to be predominantly clonal (78–82), and it is therefore not surprising that recessive deleterious or lethal alleles are found in the diploid genomes of different isolates. Indeed, clonal reproduction should fix such mutations more rapidly than sexual reproduction (83). Interestingly, LOH is frequent in *C. albicans* isolates and one might anticipate that clonal reproduction would progressively lead to homozygosity in this species. The occurrence of heterozygous SNPs affecting the function of genes with significant contributions to *C. albicans* fitness *in vivo* may contribute to the maintenance of heterozygosity if distributed on the two haplotypes of each chromosome. However, it is interesting that, under the conditions that we have used, recessive alleles with a deleterious effect *in vitro* were present only on Chr4B, with Chr4A being apparently devoid of such alleles.

## MATERIALS AND METHODS

### Strains and media.

The *C. albicans* strains used in this study are derived from SN148 (84) and are listed in Table S1 in the supplemental material. Yeast cells were grown at 30°C in liquid media in either YPD (1% yeast extract, 2% peptone, 2% dextrose) or SC (0.67% yeast nitrogen base without amino acids, 2% dextrose supplemented with the appropriate 0.08% dropout mix of amino acids). Solid media were obtained by adding 2% agar. Additionally, YPG (1% yeast extract, 2% peptone, 2% glycerol, 2% agar), sorbose-containing medium (0.7% yeast nitrogen base without amino acid, 2% l-sorbose [Fluka Analytical], 2% agarose—agarose was used instead of agar to avoid the use of scavenger cells [personal communication from Guilhem Janbon]), and 5-FOA-containing medium (0.7% yeast nitrogen base without amino acid, 0.0625% 5-fluoro-orotic acid [Toronto Research Chemicals], 0.01% uridine, 2% glucose, 2% agar, supplemented with leucine, arginine, and histidine for the needs of the experiment) have also been used.

### Plasmid and strain constructions.

We constructed a series of integrative plasmids that were sequentially introduced in the *C. albicans* strain that carries the BFP/GFP system (CEC2684; see Table S1 in the supplemental material) using the lithium acetate/polyethylene glycol protocol as previously described (85). See Text S1 for further details.

*C. albicans* transformants (see Table S1 in the supplemental material) were checked by PCR with a primer hybridizing to the plasmid sequence and a primer hybridizing to the genomic DNA (gDNA) in the region of insertion in order to verify proper integration of the plasmid in the *C. albicans* genome (see Table S2).

To facilitate reading and understanding, we named CEC4012 “I-SceI+TargetB,” CEC4088 “I-SceI+TargetA,” CEC4045 “I-SceI only,” CEC3930 “Target only,” CEC4429 “I-SceI+TargetB+*GPI16*,” and CEC4430 “I-SceI+TargetA+*GPI16*” (see Table S1 in the supplemental material)*.*

### Induction of the *Tet*-on system.

In order to activate the *Tet*-on promoter and achieve I-SceI protein overexpression, single colonies were grown overnight in SC-His-Arg medium. After 16 h of growth, the cell cultures were diluted 10 times and grown for 8 h in YPD in the presence of anhydrotetracycline at a final concentration of 3 µg/ml (ATc3) (86). The cells were then allowed to recover (i.e., to repair the DNA DSB) overnight by diluting 130 times the 8 h-grown cells in fresh YPD medium. ATc is commonly used for induction experiments and does not cause major defects in cell growth, morphology, or biology (56). The cells were diluted 50 times into 1× phosphate-buffered saline (PBS). A maximum of 10^6^ cells were analyzed by flow cytometry using a MACSQuant Analyzer (Miltenyi Biotec). The results were analyzed using FlowJo 7.6 software. The gates to determine the LOH frequencies were designed arbitrarily but remained constant for all subsequent analyses.

### Cell sorting.

Single colonies from YPD plates were cultivated as presented above. Each culture was filtered with BD Falcon cell strainers. Cells were diluted in 1× PBS at a final concentration of at least 20 × 10^6^ cells/ml. The MoFlo Astrios flow cytometer was used to analyze and sort the cells of interest at low pressure (25% to 40%) with a saline solution as a buffer (0.9% NaCl from OTEC). The flow cytometer is located at the Imagopole platform of the Institut Pasteur. A minimum of 1,000 cells were recovered, placed into 400 µl YPD in 1.5-ml sterile Eppendorf tubes, and stored at 4°C for the time of the experiment. This step can be preceded by an enrichment step consisting of sorting and recovering at a high rate the maximum number of cells first detected as positive in a 1.5-ml sterile tube and a second sorting of these enriched populations to select with a higher accuracy the truly positive cells. The sorted cells were plated immediately after cell sorting on four YPD plates and incubated at 30°C for 48 h.

Single colonies were counted and then cultivated overnight in 1 ml of fresh YPD at 30°C in 96-well plates. Aliquots were spotted on YPD, SC−Ura (SC without uracil), SC−Arg+Uri (SC without arginine supplemented with uridine), and SC−His+Uri (SC without histidine supplemented with uridine) using a 48-well or 96-well pin replicator and incubated at 30°C for 48 h. This experiment was conducted twice.

### PCRs.

Each PCR was performed in an Eppendorf Mastercycler ep gradient thermal cycler with 2 µl 10× PCR buffer; 2 µl MgCl_2_ (50 mM); 1.2 µl of a mix of deoxynucleoside triphosphates (dNTP) (2 mM); 0.5 µl (each) primer (10 µM); 0.2 µl of Taq polymerase (Invitrogen); and either 1 µl of DNA or traces of cells and water to reach a volume of 20 µl. The following conditions were used: initial denaturation at 94°C for 3 min; 30 cycles with denaturation at 94°C for 40 s, annealing at 55 or 60°C for the *MTL* locus for 40 s, and extension at 72°C for 1 min/kb; and a final extension time at 72°C for 10 min. The PCR products were verified by electrophoresis on a 1% or 2% agarose gel.

### SNP-RFLP.

Genomic DNA was extracted from cells coming from two independent cell sorting and 5-FOA experiments performed with an Epicentre kit and used as a matrix in a PCR mix with primers located upstream and downstream of the SNP(s) of interest in order to assess their heterozygous or homozygous state. We used SNP156 (TaqI restriction site) located on the left arm of Chr4, between the telomere and the BFP/GFP system, and SNP95 (AluI restriction site), located close to the centromere on the right arm of Chr4 (52). The PCRs were performed as detailed above.

### MitoTracker staining.

Cells were grown overnight in rich medium. The cultures were then diluted to an optical density at 600 nm (OD_600_) of 0.2 in 50 ml of liquid YPD and grown for 6 h at 30°C. Once an OD_600_ of 1.2 had been reached, the cells were harvested and resuspended in 10 ml of YPD. The cells were stained with MitoTracker (stock solution at 200 µM, diluted 1:1,000 in the culture) for 45 min at 30°C. The cells were washed with sterile water and resuspended in 10 ml of liquid YPG for 15 min at 30°C. The cells were fixed in 4% paraformaldehyde–1× PBS.

### Microscopy.

The fixed cells were observed with a DMR XA Leica fluorescence microscope using an oil immersion objective at ×100 magnification (1.4 numerical aperture [N/A]). Single-bandpass filters were used for Cy3 filter TX2 Leica microscopy (BP 560/40) analysis. Images were captured with an Orca II-ER cooled charge-coupled-device (CCD) camera (Hamamatsu). Cells were exposed for 250 m for the Cy3 analysis.

### 5-Fluoro-orotic acid (5-FOA) counterselection.

Single colonies were cultivated as detailed above. Dilutions of the cultures were plated on 5-FOA-containing plates (57). A total of 200,000 cells of the strains carrying both the I-SceI meganuclease and its target sequence were plated on 5-FOA-containing plates after growth in ATc-free medium, while only 2,000 and 20,000 cells, respectively, were plated after growth in the presence of ATc3. Additionally, 200,000 cells of the control strains cultured under both induced and noninduced conditions were plated on 5-FOA-containing plates. The dilutions were verified by plating a volume corresponding to 100 cells on YPD plates. Plates were incubated at 30°C for 48 h. This experiment was conducted twice.

### Sorbose counterselection.

Single colonies from two clinical strains, CEC2876 and CEC3673, along with the reference strain, SC5314, were cultivated overnight in rich medium. The cultures were diluted, and 3 × 10^7^ to 3 × 10^3^ cells were plated on sorbose-containing plates (87, 88). The dilutions were verified by plating a volume corresponding to 100 cells on YPD plates. Plates were incubated at 30°C for 10 to 12 days (88).

Single colonies were patched on sorbose plates, and a multiplex PCR at the *MLT****a***/α (89) locus was performed using primers AF120 to AF123 (see Table S2 in the supplemental material), which allow the amplification of a fragment of 821 bp for *MTL**a*** and of a fragment of 515 bp for *MTL*α.

### Whole-genome sequencing.

The genomic DNA was extracted by the use of a phenol-chloroform method. The DNA samples were prepared with a Qubit dsDNA BR assay kit following the recommendations of Thermo Fisher Scientific, and the DNA concentrations were estimated using a Qubit fluorometer.

Genomic DNAs were processed to prepare libraries for Illumina sequencing. DNA was randomly fragmented by sonication to an average fragment length of 500 bp. Illumina adapters were blunt end ligated to the fragments; a Nextera XT DNA preparation kit (Illumina) was used according to the manufacturer’s recommendations. MiSeq and HiSeq2500 platforms were used to generate, respectively, 300-bp and 250-bp paired-end reads. The sequences were mapped to *C. albicans* strain SC5314 reference genome assembly 22, available from CGD (90, 91), using BWA v0.5.9 (92). Single nucleotide polymorphisms (SNPs) between the sequenced genomes and the reference genome were identified using GATK v3.1 (93) at positions with a sequencing depth equal to or greater than 18×. Heterozygous SNPs were defined as positions where 15% or more of the calls showed one allele and 85% or less of the calls showed a second allele. Homozygous SNPs were defined as positions where more than 98% of the calls differed from the reference genome. Sequencing depth and heterozygosity/homozygosity density maps were constructed as described by Loll-Krippleber et al. (51) or Abbey et al. (94).

### Data availability.

Strains are available upon request. Table S1 in the supplemental material lists genotypes for each strain.

## SUPPLEMENTAL MATERIAL

Figure S1  Coupling of a double-strand-break-inducing system and a FACS-optimized LOH reporter system. (A) The LOH reporter system. This system consists of an artificial heterozygous locus on Chr4 with the BFP-encoding gene placed on one homolog of Chr4 and the GFP-encoding gene introduced at the same locus on the other homolog. Upon an LOH event at the BFP/GFP system integration locus, the cell can go from a doubly fluorescent state to either a mono-BFP state (LOH event on the GFP-bearing chromosome) or a mono-GFP state (LOH event on the BFP-bearing chromosome). (B) Cells undergoing an LOH event at the BFP/GFP locus are revealed by flow cytometry. On a flow cytometry output, the monofluorescent cells are localized in the side gates and the doubly fluorescent cells are found in the middle gate. (C) The DNA DSB-inducing system. The DSB-inducing system consists of (i) the gene encoding the rare-cutting endonuclease, I-SceI, placed under the control of the tetracycline-inducible promoter (P_*TET*_) integrated at the *XOG1-HOL1* locus on Chr1, (ii) the gene encoding the tetracycline-dependent reverse tetracycline-controlled transactivator (rtTA) of the P_*TET*_ promoter placed at the *ADH1* locus on Chr5, and (iii) the I-SceI target sequence integrated at the *CDR3-tG*(*GCC*)*2* locus on Chr4 on haplotype A or B between the centromere and the FACS-optimized reporter system of LOH at the *PGA59-PGA62* locus. Upon binding of anhydrotetracycline to the rtTA transactivator, the I-SceI gene is expressed and the endonuclease is directed to its target sequence, generating a DNA DSB which can be repaired by BIR/MCO in particular or, if not repaired, can experience SCL or WCL, yielding monofluorescent cells that are detected by flow cytometry. Download Figure S1, PDF file, 0.5 MB

Table S1  Strains used in this study. Table S1, PDF file, 0.1 MB

Table S2  Primers used in this study. Table S2, PDF file, 0.1 MB

Text S1  Supplemental Materials and Methods. Download Text S1, PDF file, 0.1 MB
