# Supplementary figures and images for: Analysis of Repair Mechanisms following an Induced Double-Strand Break Uncovers Recessive Deleterious Alleles in the Candida albicans Diploid Genome
Source: mBio. 2016 Oct 11;7(5):e01109-16. doi: 10.1128/mBio.01109-16 (PMC5061868; doi:10.1128/mBio.01109-16)

A

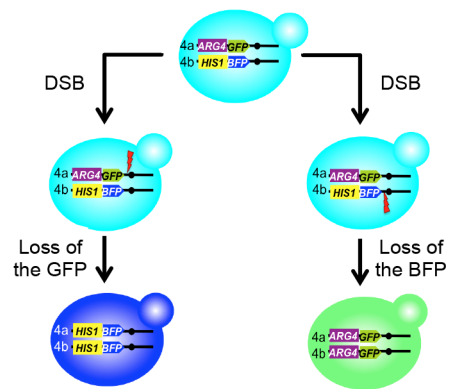

B

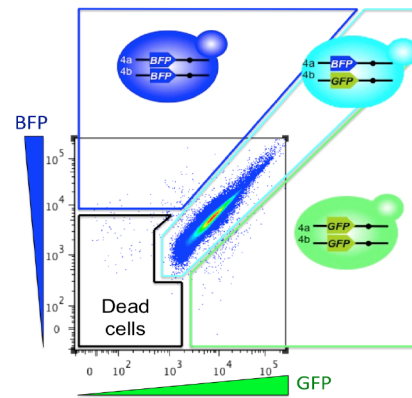

C

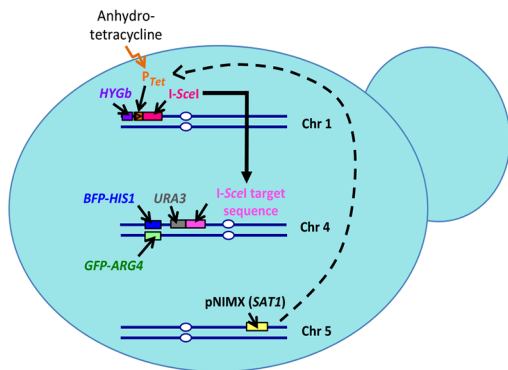

Supplement: Figure S1 — Coupling of a double-strand-break-inducing system and a FACS-optimized LOH reporter system. (A) The LOH reporter system. This system consists of an artificial heterozygous locus on Chr4 with the BFP-encoding gene placed on one homolog of Chr4 and the GFP-encoding gene introduced at the same locus on the other homolog. Upon an LOH event at the BFP/GFP system integration locus, the cell can go from a doubly fluorescent state to either a mono-BFP state (LOH event on the GFP-bearing chromosome) or a mono-GFP state (LOH event on the BFP-bearing chromosome). (B) Cells undergoing an LOH event at the BFP/GFP locus are revealed by flow cytometry. On a flow cytometry output, the monofluorescent cells are localized in the side gates and the doubly fluorescent cells are found in the middle gate. (C) The DNA DSB-inducing system. The DSB-inducing system consists of (i) the gene encoding the rare-cutting endonuclease, I-SceI, placed under the control of the tetracycline-inducible promoter (PTET) integrated at the XOG1-HOL1 locus on Chr1, (ii) the gene encoding the tetracycline-dependent reverse tetracycline-controlled transactivator (rtTA) of the PTET promoter placed at the ADH1 locus on Chr5, and (iii) the I-SceI target sequence integrated at the CDR3-tG(GCC)2 locus on Chr4 on haplotype A or B between the centromere and the FACS-optimized reporter system of LOH at the PGA59-PGA62 locus. Upon binding of anhydrotetracycline to the rtTA transactivator, the I-SceI gene is expressed and the endonuclease is directed to its target sequence, generating a DNA DSB which can be repaired by BIR/MCO in particular or, if not repaired, can experience SCL or WCL, yielding monofluorescent cells that are detected by flow cytometry. Download [file mbo005163014sf1.pdf]
