# Supplementary material for: Analysis of Repair Mechanisms following an Induced Double-Strand Break Uncovers Recessive Deleterious Alleles in the Candida albicans Diploid Genome
Source: mBio. 2016 Oct 11;7(5):e01109-16. doi: 10.1128/mBio.01109-16 (PMC5061868; doi:10.1128/mBio.01109-16)
Supplement: Table S1 — Strains used in this study. [file mbo005163014st1.pdf]

Table S1 - Strains used in this study

Strain Name	Name used in the Results section	Genotype	Auxotrophies	Parental strain	References
SN148	-	*arg4Δ/arg4Δ leu2Δ/leu2Δ his1Δ/his1Δ ura3::λimm434/ura3Δ::λimm434 iro1Δ::λimm434/iro1Δ::λimm434*	arg- his- leu- uri-	-	(1)
CEC2684	-	SN148 *Ca21chr4_C_albicans_SC5314:473390 to 476401Δ::PTDH3-GFP-CaARG4/Ca21chr4_C_albicans_SC5314:473390 to 476401Δ::PTDH3-BFP-CdHIS1*	ARG+ HIS+ leu- ura-	SN148	(2)
CEC3867	-	SN148 *Ca21chr4_C_albicans_SC5314:473390 to 476401Δ::PTDH3-GFP-CaARG4/Ca21chr4_C_albicans_SC5314:473390 to 476401Δ::PTDH3-BFP-CdHIS1 ADH1/adh1:: pNIMx*	ARG+ HIS+ leu- uri- NsnR	CEC2684	This study
CEC3888	-	*arg4Δ/arg4Δ leu2Δ/leu2Δ his1Δ/his1Δ ura3::λimm434/ura3Δ::λimm434 iro1Δ::λimm434/iro1Δ::λimm434 Ca21chr4_C_albicans_SC5314:473390 to 476401Δ::PTDH3-GFP-CaARG4/Ca21chr4_C_albicans_SC5314:473390 to 476401Δ::PTDH3-BFP-CdHIS1 ADH1/adh1:: pNIMx Ca21chr1_C_albicans_SC5314:625304 to 626436Δ::PTET-w/oGTW-HYGb-XOG1HOL1/ Ca21chr1_C_albicans_SC5314:625304 to 626436*	ARG+ HIS+ leu- uri- NsnR HygR	CEC3867	This study

CEC4021	-	*arg4Δ/arg4Δ leu2Δ/leu2Δ his1Δ/his1Δ ura3::λimm434/ura3Δ::λimm434 iro1Δ::λimm434/iro1Δ::λimm434 Ca21chr4_C_albicans_SC5314:473390 to 476401Δ::PTDH3-GFP-CaARG4/Ca21chr4_C_albicans_SC5314:473390 to 476401Δ::PTDH3-BFP-CdHIS1 ADH1/adh1:: pNIMx Ca21chr1_C_albicans_SC5314:625304 to 626436Δ::PTET-I-SceI-HYGb-XOG1HOL1/ Ca21chr1_C_albicans_SC5314:625304 to 626436*	ARG+ HIS+ leu- uri- NsnR HygR	CEC3867	This study
CEC4012	« I-*Sce*I+TargetB »	*arg4Δ/arg4Δ leu2Δ/leu2Δ his1Δ/his1Δ ura3::λimm434/ura3Δ::λimm434 iro1Δ::λimm434/iro1Δ::λimm434 Ca21chr4_C_albicans_SC5314:473390 to 476401Δ::PTDH3-GFP-CaARG4/Ca21chr4_C_albicans_SC5314:473390 to 476401Δ::PTDH3-BFP-CdHIS1 ADH1/adh1:: pNIMx Ca21chr1_C_albicans_SC5314:625304 to 626436Δ:: PTET-I-SceI-HYGb-XOG1HOL1/ Ca21chr1_C_albicans_SC5314:625304 to 626436 Ca21chr4_C_albicans_SC5314:775939 to 779223Δ::pFA-ISceI_TS-URA3-CDR3-tG(GCC)2/ Ca21chr4_C_albicans_SC5314:775939 to 779223*	ARG+ HIS+ leu- URI+ NsnR HygR	CEC4021	This study

CEC4088	« I-*Sce*I+TargetA »	*arg4Δ/arg4Δ leu2Δ/leu2Δ his1Δ/his1Δ ura3::λimm434/ura3Δ::λimm434 iro1Δ::λimm434/iro1Δ::λimm434 Ca21chr4_C_albicans_SC5314:473390 to 476401Δ::PTDH3-GFP-CaARG4/ Ca21chr4_C_albicans_SC5314:473390 to 476401Δ::PTDH3-BFP-CdHIS1 ADH1/adh1::pNIMx Ca21chr1_C_albicans_SC5314:625304 to 626436Δ::PTET-I-SceI-HYGb-XOG1HOL1/ Ca21chr1_C_albicans_SC5314:625304 to 626436 Ca21chr4_C_albicans_SC5314:775939 to 779223Δ:: pFA-ISceI_TS-URA3-CDR3-tG(GCC)2/ Ca21chr4_C_albicans_SC5314:775939 to 779223*	ARG+ HIS+ leu- URI+ NsnR HygR	CEC4021	This study
CEC4045	« I-*Sce*I only»	*arg4Δ/arg4Δ leu2Δ/leu2Δ his1Δ/his1Δ ura3::λimm434/ura3Δ::λimm434 iro1Δ::λimm434/iro1Δ::λimm434 Ca21chr4_C_albicans_SC5314:473390 to 476401Δ::PTDH3-GFP-CaARG4/Ca21chr4_C_albicans_SC5314:473390 to 476401Δ::PTDH3-BFP-CdHIS1 ADH1/adh1::pNIMx Ca21chr1_C_albicans_SC5314:625304 to 626436Δ:: PTET-I-SceI-HYGb-XOG1HOL1/Ca21chr1_C_albicans_SC5314:625304 to 626436 RPS1/rps1::*CIp10	ARG+ HIS+ leu- URI+ NsnR HygR	CEC4021	This study

CEC3930	« Target only»	*arg4Δ/arg4Δ leu2Δ/leu2Δ his1Δ/his1Δ ura3::λimm434/ura3Δ::λimm434 iro1Δ::λimm434/iro1Δ::λimm434 Ca21chr4_C_albicans_SC5314:473390 to 476401Δ::PTDH3-GFP-CaARG4/Ca21chr4_C_albicans_SC5314:473390 to 476401Δ::PTDH3-BFP-CdHIS1 ADH1/adh1:: pNIMx Ca21chr1_C_albicans_SC5314:625304 to 626436Δ:: PTET-w/oGTW-HYGb-XOG1HOL1/ Ca21chr1_C_albicans_SC5314:625304 to 626436 Ca21chr4_C_albicans_SC5314:775939 to 779223Δ:: pFA-ISceI_TS-URA3-CDR3-tG(GCC)2/Ca21chr4_C_albicans_SC5314:775939 to 779223*	ARG+ HIS+ leu- URI+ NsnR HygR	CEC3888	This study
CEC4429	« I-*Sce*I+TargetB+*GPI16*»	*arg4Δ/arg4Δ leu2Δ/leu2Δ his1Δ/his1Δ ura3::λimm434/ura3Δ::λimm434 iro1Δ::λimm434/iro1Δ::λimm434 Ca21chr4_C_albicans_SC5314:473390 to 476401Δ::PTDH3-GFP-CaARG4/Ca21chr4_C_albicans_SC5314:473390 to 476401Δ::PTDH3-BFP-CdHIS1/Ca21chr4_C_albicans_SC5314:775939 to 779223Δ::pFA-ISceI_TS-URA3-CDR3-tG(GCC)2/Ca21chr4_C_albicans_SC5314:775939 to 779223 ADH1/adh1:: pNIMx Ca21chr1_C_albicans_SC5314:125966 to 131098Δ::PTET-I-SceI-HYGb-XOG1HOL1/Ca21chr1_C_albicans_SC5314:625304 to 626436 RPS1/rps1::PTDH3-C4_03130W_A-CdLEU2*	Prototroph NsnR HygR	CEC4012	This study

CEC4430	« I-*Sce*I+TargetA+*GPI16*»	*arg4Δ/arg4Δ leu2Δ/leu2Δ his1Δ/his1Δ ura3::λimm434/ura3Δ::λimm434 iro1Δ::λimm434/iro1Δ::λimm434 Ca21chr4_C_albicans_SC5314:473390 to 476401Δ::PTDH3-GFP-CaARG4/Ca21chr4_C_albicans_SC5314:473390 to 476401Δ::PTDH3-BFP-CdHIS1/Ca21chr4_C_albicans_SC5314:775939 to 779223Δ::pFA-ISceI_TS-URA3-CDR3-tG(GCC)2/Ca21chr4_C_albicans_SC5314:775939 to 779223 ADH1/adh1:: pNIMx Ca21chr1_C_albicans_SC5314:125966 to 131098Δ:: PTET-I-SceI-HYGb-XOG1HOL1/Ca21chr1_C_albicans_SC5314:625304 to 626436 RPS1/rps1::PTDH3-C4_03130W_A-CdLEU2*	Prototroph NsnR HygR	CEC4088	This study
CEC4797	« Small+P$_{TDH3}$-*MRF2* »	*arg4Δ/arg4Δ leu2Δ/leu2Δ his1Δ/his1Δ ura3::λimm434/ura3Δ::λimm434 iro1Δ::λimm434/iro1Δ::λimm434 Ca21chr4_C_albicans_SC5314:473390 to 476401Δ::PTDH3-GFP-CaARG4/Ca21chr4_C_albicans_SC5314:473390 to 476401Δ::PTDH3-BFP-CdHIS1 ADH1/adh1:: pNIMx Ca21chr1_C_albicans_SC5314:125966 to 131098Δ:: PTET-I-SceI-HYGb-XOG1HOL1/Ca21chr1_C_albicans_SC5314:625304 to 626436 rps1::PTDH3-C4_03130W_A-CdLEU2/rps1::PTDH3-C4_03750C_A-CaURA3*	Prototroph NsnR HygR	CEC4430	This study

CEC4798	« Small+P_{MRF2}-*MRF2* »	*arg4Δ/arg4Δ leu2Δ/leu2Δ his1Δ/his1Δ ura3::λimm434/ura3Δ::λimm434 iro1Δ::λimm434/iro1Δ::λimm434 Ca21chr4_C_albicans_SC5314:473390 to 476401Δ::PTDH3-GFP-CaARG4/Ca21chr4_C_albicans_SC5314:473390 to 476401Δ::PTDH3-BFP-CdHIS1 ADH1/adh1:: pNIMx Ca21chr1_C_albicans_SC5314:125966 to 131098Δ:: PTET-I-SceI-HYGb-XOG1HOL1/Ca21chr1_C_albicans_SC5314:625304 to 626436 rps1::PTDH3-C4_03130W_A-CdLEU2/rps1::PMRF2-C4_03750C_A-CaURA3*	Prototroph NsnR HygR	CEC4430	This study
CEC4817	« Small+*URA3*»	*arg4Δ/arg4Δ leu2Δ/leu2Δ his1Δ/his1Δ ura3::λimm434/ura3Δ::λimm434 iro1Δ::λimm434/iro1Δ::λimm434 Ca21chr4_C_albicans_SC5314:473390 to 476401Δ::PTDH3-GFP-CaARG4/Ca21chr4_C_albicans_SC5314:473390 to 476401Δ::PTDH3-BFP-CdHIS1 ADH1/adh1:: pNIMx Ca21chr1_C_albicans_SC5314:125966 to 131098Δ:: PTET-I-SceI-HYGb-XOG1HOL1/Ca21chr1_C_albicans_SC5314:625304 to 626436 rps1::PTDH3-C4_03130W_A-CdLEU2/rps1::*CIp10	Prototroph NsnR HygR	CEC4430	This study
CEC2876	CEC2876	CLINICAL ISOLATE	Prototroph	-	(M.E.B., G.S., N.S., K.S., C.M., and C.d'E., manuscript *in preparation*)

CEC3673	CEC3673	CLINICAL ISOLATE	Prototroph	-	(M.E.B., G.S., N.S., K.S., C.M., and C.d'E., manuscript *in preparation*)
SC5314	SC5314	Reference strain	Prototroph	-	(3)

(1) Noble SM, Johnson AD: Strains and strategies for large-scale gene deletion studies of the diploid human fungal pathogen *Candida albicans*. Eukaryot Cell 2005, 4:298-309.
(2) Loll-Krippleber R *et al*.: A study of the DNA damage checkpoint in *Candida albicans*: uncoupling of the functions of Rad53 in DNA repair, cell cycle regulation and genotoxic stress-induced polarized growth. Mol Microbiol 2014, 91:452-471.
(3) Fonzi WA, Irwin MY: Isogenic strain construction and gene mapping in *Candida albicans*. Genetics 1993, 134:717-728.
