# Supplementary material for: Analysis of Repair Mechanisms following an Induced Double-Strand Break Uncovers Recessive Deleterious Alleles in the Candida albicans Diploid Genome
Source: mBio. 2016 Oct 11;7(5):e01109-16. doi: 10.1128/mBio.01109-16 (PMC5061868; doi:10.1128/mBio.01109-16)
Supplement: Table S2 — Primers used in this study. [file mbo005163014st2.pdf]

Table S2 - Primers used in this study

	Primer names	Sequence
PCR amplification for cloning	AF001	gggaaagccggcGACAATCACGAAGCCAAGTAAG
	AF002	aaaggggccggcTTACCGCTCGGCTTTGTTCC
	AF006	gggaaagttaacGAAGGAAGATGAATTGGGTTGC
	AF007	aaagggccgcggGGGAGTCTCTCGTGAAGAATATG
	AF008	gggaaactgcagattaccctgttatccctaGGGTGGTCATACTATGTGTTGTTG
	AF009	aaagggaagcttAGGAGACTACAAACTGGAAGG
	AF027	cccaaagatatcATGccaccaaaaaaaaaaagaaaagttcataaaaatattaaaaaaaatcaagtt
	AF031	aaagggatgcatttattttaaaaaagtttctgatgaaatagt
	AF049	AAACCCgatatcATGCATTTGTTACTAAGGATACC
	AF050	CCCAAAacgcgtgccggcCTATTGTGATCTATAAACATCGATC
	AF113	CTCGAGACCGGTggagagttcttaatacttga
	AF114	CAGCTGgtggtaaggtgctaatctaa
PCR MTLa/MTLα	AF120	TTGAAGCGTGAGAGGCAGGAG
	AF121	GTTTGGGTTCCTTCTTTCTCATTC
	AF122	TTCGAGTACATTCTGGTCGCG
	AF123	TGTAAACATCCTCAATTGTACCCGA
PCR amplification to check the integration	XOGHOL-verif F	GCTGTCATCTACTGGTTTGTG
	XOGHOL-verif R	GTGCGTTGGAACACCAGTTG
	Verif-CDR3-F	TGGTGGGTAAAGGGCATATTC
	Verif-TG(GCC)2-R	GTTGGTATCATGGTGATGTGTC
	CIpUR	ATTACTATTTACAATCAAAGGTGGTC
	CIpUL	ATACTACTGAAAATTTCCTGACTTTC
	CIpUL_2	AGATACTCACGCACGCCCATACTACT
	Leu2_down	GCTACTGAAGTTGGTGACGCGATTGT
	ADH1verif	ACAAGCTCATTGAGTGACGAAAAG
	PNIM1verif	tttacgggttgttaaaccttcgat
	URA3_rev_AF	gttgtcctaatccatcacct
	URA3_fwd_AF	aactcatgcctcaccagtag

SNP-RFLP	SNP-95-F#2	CATGCCCGCTTGAAACTACC
	SNP-95-R#2	GTCAGTGATTCAGTTGAAGTGG
	SNP 156-R	TGGGTTTGGACATCAGGTTCAA
	SNP-156-F#2	ACAGAACAGTAGATTCCAAC
	SNP-23-F	AGCCAACCATATTTCAGGATTGAC
	SNP-23-R	GTGCCAACTAGTAATGGTTGTCAT
	SNP-42-F	GTACTTCTATACACGCACATCTTCA
	SNP-42-R	GAAATCCACCGCATAAGAAATGGTT
