# Supplementary material for: Analysis of Repair Mechanisms following an Induced Double-Strand Break Uncovers Recessive Deleterious Alleles in the Candida albicans Diploid Genome
Source: mBio. 2016 Oct 11;7(5):e01109-16. doi: 10.1128/mBio.01109-16 (PMC5061868; doi:10.1128/mBio.01109-16)
Supplement: Text S1 — Supplemental Materials and Methods. Download [file mbo005163014s1.pdf]

Text S1 – Supplementary Material and Methods

Plasmid constructions

p*Tet*-I-*Sce*I-*HYGb-XOG1HOL1* harbors the I-*Sce*I gene under the control of a tetracycline inducible promoter (56), the hygromycin B resistance gene (88) and a sequence that allows its integration at the *XOG1-HOL1* intergenic region on chromosome 1 (Chr1). To generate this plasmid, CIpOp2, a CIp10 derivative containing the *Tet*-on promoter (56) was digested with *Eco*RV to excise the Gateway® Cassette and allowed to religate (p*Tet*-w/oGTW). The resulting plasmid was digested with *Not*I to excise the *URA3* marker and the *RPS1* sequences and recircularized, yielding p*Tet*-w/oGTW-w/o*URA3*. The *HYGb* gene was excised from pKS-*ACT1*p-*HYGb* (88) by *Xba*I and *Acc*65I, blunt-ended with the Klenow enzyme (Invitrogen™) and cloned into the *Ale*I-linearized p*Tet*-w/oGTW-w/o*URA3* plasmid, yielding p*Tet*-w/oGTW-*HYGb*. Then, a 1kb sequence from the intergenic region between *XOG1* and *HOL1* genes was amplified by PCR with oligonucleotides AF1 and AF2 (Table S2). The PCR product was digested with *Nae*I and ligated into the *Nae*I-linearized p*Tet*-w/oGTW-*HYGb* plasmid yielding p*Tet*-w/oGTW-*HYGb-XOG1HOL1*. Finally, as *C. albicans* is part of the CTG clade (89), we used http://genomes.urv.es/OPTIMIZER/ website (90) to optimize the I-*Sce*I meganuclease gene sequence to the *C. albicans* codon usage . The optimized I-*Sce*I gene flanked by *Nsi*I and *Eco*RV restriction sites was synthesized by GeneArt® Invitrogen™, and cloned into the p*Tet*-w/oGTW-*HYGb-XOG1HOL1* plasmid linearized with *Nsi*I and *Eco*RV to yield p*Tet*-I-*Sce*I-*HYGb-XOG1HOL1*.

p*Tet*-NLS-I-*Sce*I-*HYGb-XOG1HOL1* was constructed by using the Nuclear Localization Sequence (NLS) from SV40 (91) carrying a region recognized by Importin α (92) and which has already been used in *C. albicans* (93). We used the http://genomes.urv.es/OPTIMIZER/ website to optimize the codon usage for *C. albicans* as follow: ATG CCA CCA AAA AAA AAA AGA AAA GTT CAT. We fused this modified NLS sequence to I-*Sce*I by PCR using

primers AF27 and AF31 (Table S2), and p*Tet*-I-*Sce*I-*HYGb-XOG1HOL1* as a template. The PCR fragment was cloned into a TOPO®-TA vector (Thermofisher), digested with *Eco*RV and *Nsi*I and cloned into p*Tet*-I-*Sce*I-*HYGb-XOG1HOL1* digested with the same enzymes, yielding p*Tet*-NLS-I-*Sce*I-*HYGb-XOG1HOL1.*

pFA-I-*Sce*I-TS-*URA3-CDR3/tG(GCC)2* carries the I-*Sce*I recognition sequence, the *URA3* marker and two sequences that allow integration in the *CDR3/tG(GCC)*$_2$ intergenic region on Chr4. To obtain this plasmid, we first amplified two sequences in the intergenic region between *CDR3* and *tG(GCC)2* genes. The first region at position 775,906-776,737, closer to *CDR3*, is 831bp long and was amplified by PCR with oligonucleotides AF6 and AF7 (Table S2). The second region at position 778,136-779,189, closer to *tG(GCC)*$_2$*,* is 1,055bp long and was amplified by PCR with oligonucleotides AF8 and AF9, containing the I-*Sce*I target sequence (attaccctgttatccctа, Table S2**)**. The *Sac*II+*Hpa*I-digested AF6/AF7 PCR product was inserted at the *Sac*II and *Hpa*I sites of pFA-*CaURA3* (94), carrying the *URA3* gene. The resulting plasmid was then double-digested with *Hind*III-*Pst*I to insert the *Hind*III+*Pst*I-cut AF8/AF9 PCR product. The resulting plasmid was named pFA-I-*Sce*I-TS-*URA3-CDR3/tG(GCC)2*.

The p*CaTDH3*-GTW-*LEU2* plasmid carries the sequence for integration at the *RPS1* locus on Chr1, the *C. maltosa* optimized *LEU2* marker and a Gateway® Cassette flanked by the *attR* sequences and under the control of the P_{TDH3} constitutive promoter. To obtain this plasmid, the vector pFA-*CmLEU2* (95) was digested with *Pvu*II and *Spe*I, the band corresponding to the *LEU2* marker was purified and cloned into the *Ale*I-*Spe*I double-digested p*CaTDH3*-GTW-*URA3* plasmid (51). The resulting plasmid was called p*CaTDH3*-GTW-*LEU2.*

p*CaTDH3-GPI16-LEU2* was constructed by using the Gateway® Technology as described in Chauvel *et al.* (56) by LR recombination between the BP vector containing orf19.2677 (*GPI16*) and the destination vector pCa*TDH3*-GTW-*LEU2*.

p*CaTDH3-MRF2-URA3* was constructed by cloning orf19.1303 (*MRF2*) under the control of a constitutive promoter in p*CaTDH3*-GTW-*URA3*, a CIpOp2 derivative plasmid (51). This plasmid was digested with *Bsr*GI to excise the Gateway® Cassette and allowed to religate, resulting in p*CaTDH3-URA3*. orf19.1303 was amplified by PCR using primers AF49 and AF50 (Table S2) containing *Eco*RV and *Mlu*I-*Nae*I restriction sites at the 3' and 5' ends, respectively. The PCR amplified DNA was gel purified and cloned into a TOPO®-TA vector. Sequencing was performed to select the TOPO-TA-*MRF2* clone carrying the full-length allele. The *MRF2* gene was excised from the TOPO-TA-*MRF2* vector by *Eco*RV-*Mlu*I digestion and cloned into the p*CaTDH3-URA3* plasmid linearized with *Eco*RV-*Mlu*I, yielding p*CaTDH3-MRF2-URA3*.

p*CaMRF2-MRF2-URA3* was obtained by replacing the P_{TDH3} promoter from p*CaTDH3-MRF2-URA3* by the endogenous *MRF2* promoter. The promoter of *MRF2* was PCR amplified with AF113 and AF114 (Table S2) containing *Eco*RV and *Mlu*I-*Nae*I restriction sites at the 3' and 5' ends. The PCR product was gel purified and cloned into a TOPO®-TA vector. The *MRF2* promoter was excised from the TOPO®-TA-p*CaMRF2* vector by *Xho*I, *Pvu*II and *Dra*III digestion and cloned into the *Xho*I-*Eco*RV double-digested p*CaTDH3-MRF2-URA3* plasmid to replace the P_{TDH3} promoter yielding p*CaMRF2-MRF2-URA3.*

Strain constructions

CEC2684 was first transformed with *Sac*II-*Kpn*I-digested pNIMX (56) to introduce the transactivator for the *Tet*-on promoter (56). pNIMX (56) is a pNIM1 derivative (96) and integrates at the *ADH1* locus on Chr5. Transformants were selected on nourseothricin-containing media (300µg/mL) (97) and validated by PCR with the ADH1verif and NIM1verif primers (Table S2), giving rise to CEC3867. CEC3867 was then transformed with the *Pml*I-linearized p*Tet*-NLS-*I-SceI-HYGb-XOG1HOL1* or p*Tet*-w/oGTW-*HYGb-XOG1HOL1*

plasmids, which integrate at the *XOG1-HOL1* intergenic region of Chr1. After selection on hygromycin B-containing plates (700µg/mL) (88), we obtained CEC4021 and CEC3888, respectively. Finally, CEC4021 was transformed with *Nco*I-*Hind*III-digested pFA-I-*Sce*I-TS-*URA3-CDR3/tG(GCC)2*, integrating at the *CDR3-tG(GCC)2* intergenic region, on Chr4, located 302,518Kb away from the *PGA59-PGA62* locus and 214,361Kb away from the centromere, yielding the CEC4012 ("I-*Sce*I + TargetB") or CEC4088 ("I-*Sce*I + TargetA") strains. We also constructed a first control strain that carries only the inducible *I-SceI* gene, but no I-*Sce*I recognition sequence. Because of uridine auxotrophy, this control strains was transformed with the *Stu*I-digested CIp10 plasmid (98) that integrates at the *RPS1* locus on Chr1 yielding CEC4045 ("I-*Sce*I only"). The second control strain, carrying only the I-*Sce*I target sequence but no I-*Sce*I gene, CEC3930 ("Target only"), was obtained by transforming CEC3888 with the pFA-I-*Sce*I-TS-*URA3-CDR3/tG(GCC)2* construction.

To obtain the strain overexpressing *GPI16* with or without the *MRF2* gene, we first transformed CEC4012 and CEC4088 with p*CaTDH3-GPI16-LEU2*, yielding CEC4429 and CEC4430 respectively. Subsequently, a small colony derived from CEC4430 having lost the I-*Sce*I target sequence-bearing chromosome was transformed with the p*CaTDH3-MRF2-URA3* or p*CaMRF2-MRF2-LEU2* plasmids, leading to CEC4797 and CEC4798.
